# Supplementary material for: The Putative Cytochrome b5 Domain-Containing Protein CaDap1 Homologue Is Involved in Antifungal Drug Tolerance, Cell Wall Chitin Maintenance, and Virulence in Candida albicans
Source: J Fungi (Basel). 2024 Apr 26;10(5):316. doi: 10.3390/jof10050316 (PMC11122062; doi:10.3390/jof10050316)
Supplement: Supplementary file 1 [file jof-10-00316-s001.zip › jof-2940499-supplementary.pdf]

Supplemental file

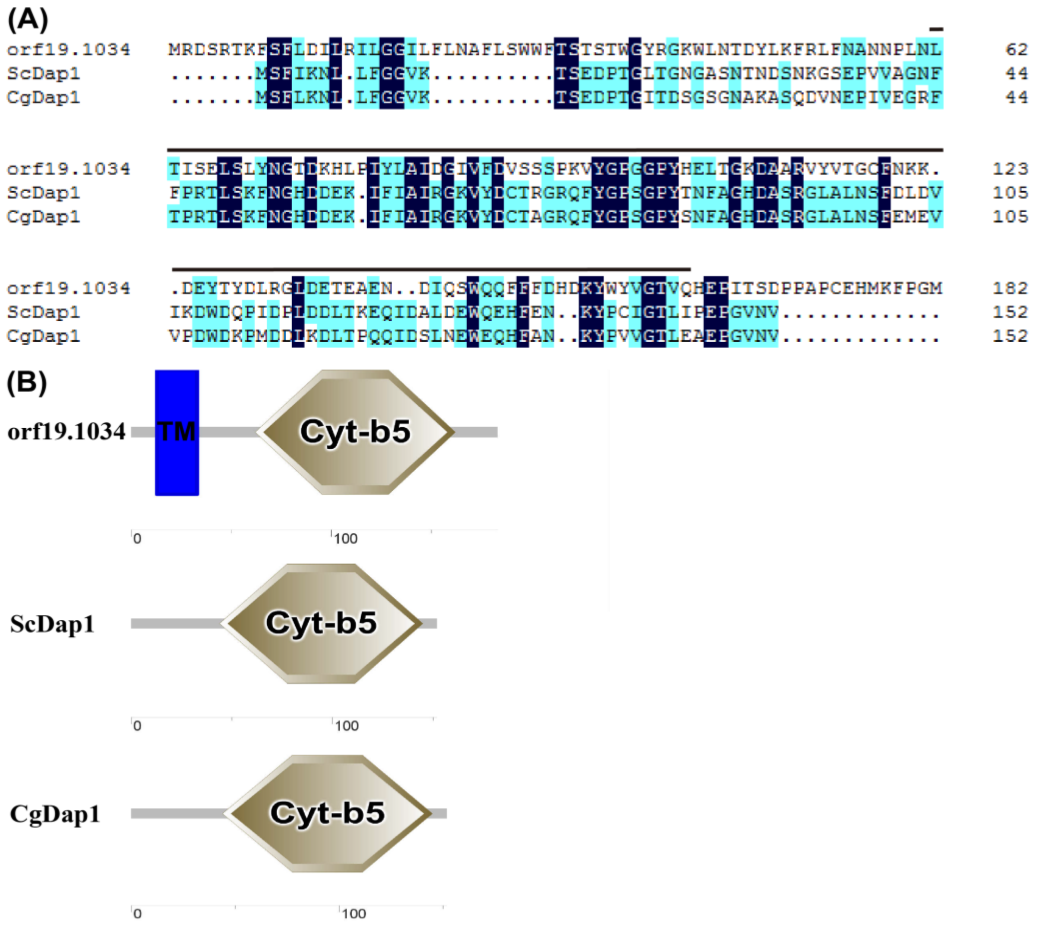

Figure S1. Predicted yeast Dap1 ortholog, CaDap1 (orf19.1034) in *Candida albicans*. (A) Amino acid sequence alignment of *C. albicans* CaDap1 (orf19.1034) and its homologous sequences from *Saccharomyces cerevisiae* (ScDap1) and *C. glabrata* (CgDap1). The horizontal line represents the predicted cytochrome *b*5-like heme-binding domain of the CaDap1 (orf19.1034). (B) Predicted cytochrome *b*5-like heme-binding and transmembrane (TM) domains in CaDap1 (orf19.1034), ScDap1, and CgDap1, based on a SMART protein search (http://smart.embl-heidelberg.de/).

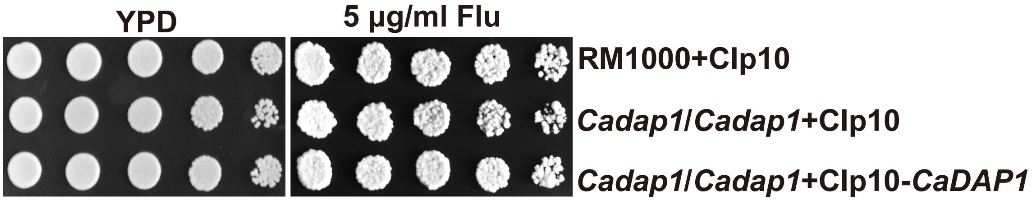

Figure S2. The *Cadap1/Cadap1* mutant exhibited insensitive to fluconazole (Flu). Growth phenotypes of the WT, *Cadap1/Cadap1* mutant, and *CaDAP1* RS were evaluated following exposure to 5 μg/ml fluconazole. The cells cultured overnight were renewed in YPD medium and grown to exponential phase at 30°C. Following this, they underwent a tenfold serial dilution and were then spotted sequentially onto plates supplemented with fluconazole. Phenotypic characteristics were examined following incubation at 30°C for 48-72 hours.

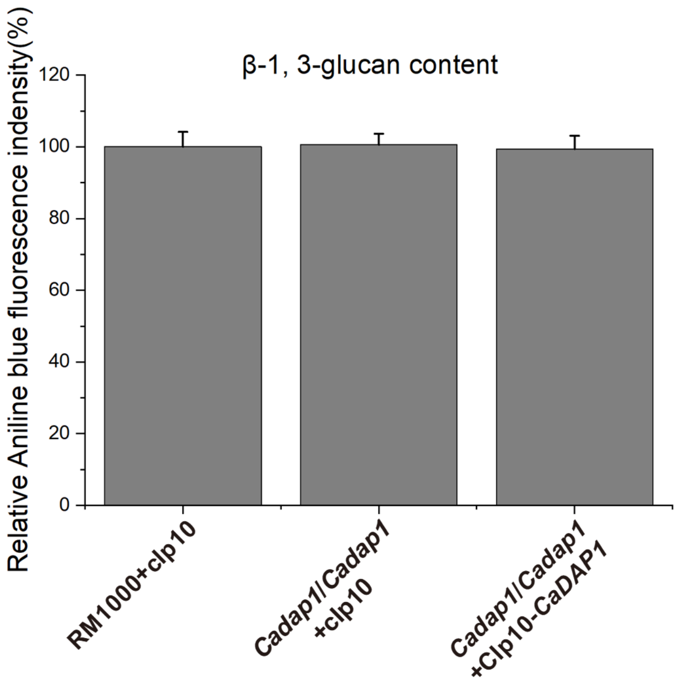

Figure S3. Deletion of *CaDAP1* leads to unchanged β-1,3-glucan content in cell walls.
